# Mesothelioma Driver Genes, Ferroptosis, and Therapy

**DOI:** 10.3389/fonc.2019.01318

**Published:** 2019-11-27

**Authors:** Emanuela Felley-Bosco, Steven G. Gray

**Affiliations:** ^1^Laboratory of Molecular Oncology, Department of Thoracic Surgery, University Hospital Zurich, Zurich, Switzerland; ^2^Thoracic Oncology Research Group, Trinity Translational Medical Institute, St. James's Hospital, Dublin, Ireland

**Keywords:** mesothelioma, ferroptosis, regulated cell death, NF2, BAP1

If a given cell has a propensity to die in a certain manner, the logical step for this cell to become a cancer cell is to insure its survival by installing mechanisms circumventing the predestined regulated cell death. A clear example of this occurs in follicular lymphoma where chromosomal re-arrangements result in Bcl2 overexpression, allowing escape from apoptosis and tolerance to undesired generation of otherwise physiological mutations and double strand breaks necessary to produce the variability necessary for antigen recognition site by immunoglobulin (1).

The predestined regulated cell death mechanism for mesothelial cells is not known, but recent data have linked two frequent drivers of mesothelioma, *NF2* and *BAP1* (2, 3), to ferroptosis (4, 5). The latter is a more recently described type of iron-dependent regulated cell death (6).

An additional driver of mesothelioma, which is however less specific to this cancer type, is loss of *CDKN2A* (7–9). One of the products encoded by *CDKN2A* gene is p16, which is one of the effectors of senescence (10). The latter is a state of stable cell cycle arrest with active metabolism where resistance to ferroptosis induction has been observed due to decreased iron bioavailability, linked to increased ferritin (*FTH1*) levels, and accompanied by increased levels of iron regulatory protein 2 (*IREB2*) and decreased levels of iron-cluster assembly enzyme (*ISCU*) (11).

The aim of this Opinion paper is to complement the editorial by Fennell (12) with some additional considerations, which include potential ideas regarding treatment, based on data from our own model of mesothelioma development (13) and the mesothelioma TCGA database (3).

In ferroptosis (Figure 1A), cell death is executed by reactive oxygen species (ROS)-mediated peroxidation of polyunsaturated fatty acids (PUFAs). The origin of ROS includes incomplete reduction of oxygen during electron transport to form superoxide, and a direct generation of superoxide by the membrane bound NADPH oxidases (NOX) (14). Lipid peroxidation is prevented by glutathione peroxidase 4 (GPX4), which uses glutathione (GSH) as reducing agent [reviewed in (15)]. GSH is synthesized from cysteine, which is either derived from methionine through methionine-R-sulfide reductase B2 (MSRB2), or it is imported. Interestingly, *MRSB2* expression is significantly higher in epithelioid compared to tissues with a sarcomatoid molecular profile (2). Import of cysteine is mediated by SLC7A10 transporter, but cysteine can also be derived from the reduction of cystine (product of the oxidation of two cysteine molecules, which are then linked via a disulfide bond). Cystine is transported into the cell through the system Xc^−^ transporter, which includes SLC7A11 subunit. It is worth noting that only cystine is present in cell culture medium, and, as for cells like lymphocytes [reviewed in (16)], mesothelial and mesothelioma primary cells grow better in the presence of beta-mercaptoethanol (17, 18). This effect is likely due to formation of beta-mercaptoethanol dimers with cystine facilitating its uptake by other transporters (19).

**Figure 1:**
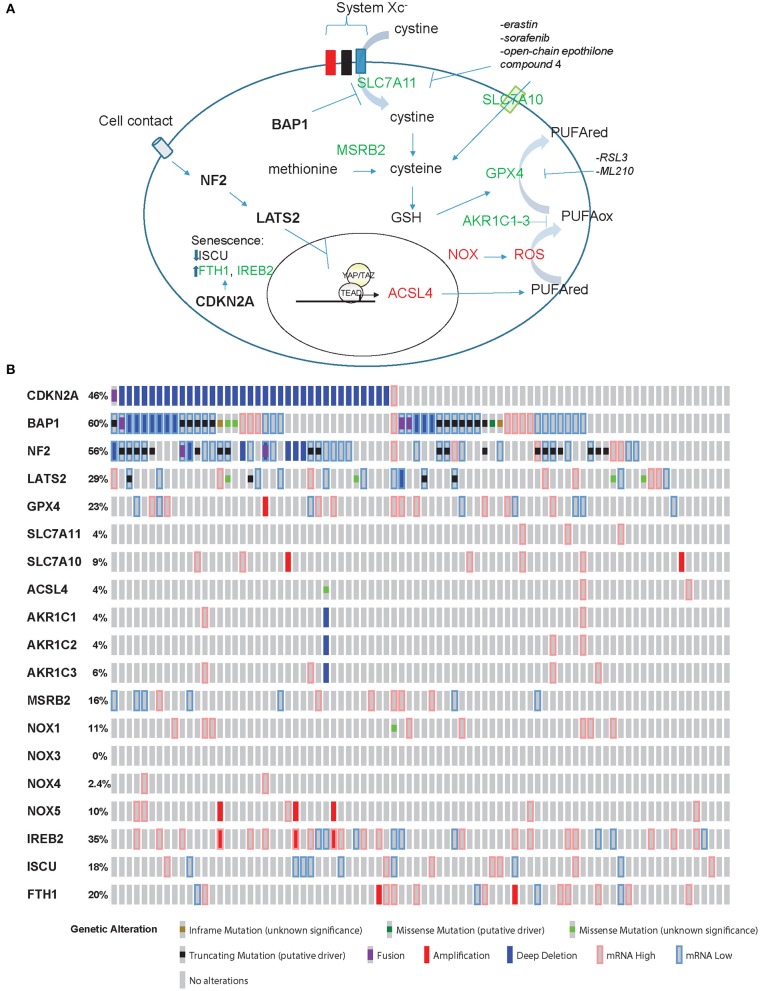
Ferroptosis effectors in mesothelioma. **(A)** Model for ferroptosis pathway. Promoters of ferroptosis (red) include ACSL4, NOX, and ROS, while SLC7A11, SLC7A10, MSRB2, GPX4, and AKR1C1-3 (green) are ferroptosis scavengers. *ACSL4* expression is activated by YAP/TAZ while BAP1 inhibits the expression of *SLC7A11*. CDKN2A-encoded p16 is one of the effectors of senescence where ferroptosis is prevented by increased expression of FTH1 and IREB2 accompanied by decreased levels of ISCU. **(B)** “Oncoprint” analysis of ferroptosis effectors in TCGA data performed using cBioportal (www.cBioportal.org).

BAP1 decreases the expression of SLC7A11 (5), leading to increased sensitivity to ROS and erastin in mesothelioma cells.

PUFA abundance, and hence predisposition to ferroptosis, is dependent on the expression of acyl-CoA synthetase long-chain family member 4 (ACSL4). In the absence of a negative control downstream NF2/Hippo pathway, the transcriptional co-activator YAP increases *ACSL4* expression (4). Resistance to ferroptosis is associated with high expression levels of *aldo-keto reductase 1-3(AKR1C1-3)* (20). These enzymes have been shown to participate in the detoxification of reactive aldehyde generated downstream of the oxidation of various PUFA.

Taking into account all this information, a mesothelial cell losing BAP1 function becomes resistant to ROS and ferroptosis, while mesothelial cells losing NF2 function become “primed” for ferroptosis, while loss of p16 expression will be associated with impaired senescence-driven ferroptosis resistance.

Loss of BAP1 is mostly associated with epithelioid histotype (21), while loss of NF2 function is mostly associated with high S-score, which identifies tumor samples with a high sarcomatoid phenotype component (22). This is consistent with the observation that cells in a mesenchymal state, which are less sensitive to chemotherapeutics, have been shown to rely on GPX4 function to avoid ferroptosis (23–25). Intriguingly, Nagai et al. observed that iron chelation did not prevent mesothelioma development in rats upon exposure to asbestos fibers, but tumor histotype shifted toward increased incidence of epithelioid compared to the sarcomatoid histotype observed in the control group (26). In the absence of accompanying genomic alteration analysis of those tumors it is not possible to know whether the two groups had a different genetic alteration profile or whether there was a plasticity response of cancer cells to the environment.

Recently, in our own model of mesothelioma development (13) we observed a significant (*p* = 0.008971, FDR = 0.0145) 1.4-fold increase of *Acsl4* and a significant 74 and 91% decrease of *Gpx4* (*p* = 6.28E-22, FDR = 8.19E-21) and *Msrb2* (*p* = 1.38E-88, FDR = 4.95E-86) expression, respectively, when comparing tumors to inflamed precancerous lesions. Hence, these tumors should be predisposed to ferroptosis death, as expected from their spindeloid phenotype and YAP activation. However, *Slc7a11* undergoes a significant (*p* = 0.004227, FDR = 0.007263) 4.7-fold upregulation as well, consistent with the loss of one BAP1 allele. Collectively, these observations suggest that tumors with alterations in both pathways, NF2 and BAP1, which occur in a significant fraction of MPM patients according to TCGA data (3) (Figure 1B), might be more resistant to ferroptosis. However, functional studies are necessary to verify this hypothesis.

Drugs modulating ferroptosis have been recently reviewed (27). Inhibitors of GPX4, such as Ras-selective-lethal 3 (RSL3) or ML210, trigger ferroptosis, while SLC7A11 inhibiting agents, such as erastine or sorafenib, lead to glutathione depletion and endoplasmic reticulum stress. The mechanism behind sorafetinib inhibition of cysteine Xc^−^ transporter is not clear and is possibly indirect (20). Dr. Fennell pointed to two clinical trials in mesothelioma (28, 29), where sorafenib was used and in which objective responses were observed in only in a small proportion of unselected patients. Therefore, it will be necessary to have a translational study accompanying these trials to determine if those patients that responded had a disrupted NF2/Hippo pathway.

Relevant for the current first-line therapy of mesothelioma patients, which includes cisplatin, erastin has been shown to have a synergistic cancer cell killing effect with cisplatin in *in vitro* models (30).

Remarkably, in a recent study ferroptosis was observed in cells treated with some open-chain epothilones small molecules in a manner similar to that of erastin (25). Additionally, mesothelioma cell killing is iron-dependent in a novel therapeutic approach using atmospheric plasma therapy (31). Plasma is the fourth condition of physical state, in addition to solid/liquid/gas [reviewed in (32)].

Given the propensity of mesenchymal cells to be sensitive to ferroptosis induction, it is tempting to suggest that mesothelioma patients with high S-score might benefit from this novel therapy. However, a plethora of novel therapies for mesothelioma have emerged (33–35) and it might be worth assessing whether mesothelioma cells can undergo ferroptosis *in vivo*. Indeed, it must be noted that Carbonic anhydrase 9 (CAIX) has recently been shown to confer resistance to ferroptosis/apoptosis in malignant mesothelioma under hypoxia (36). Given that CAIX is ubiquitously highly expressed in mesothelioma (37, 38), this may have to be taken into account moving forwards. Because of the known effect of cisplatin on ROS generation [reviewed in (39)], it may also be of use to analyze the expression of *PTSG2*, encoding for COX-2, a marker of ferroptosis (40), in samples from these cisplatin-treated patients.

## Author Contributions

EF-B and SG wrote and approved the manuscript.

### Conflict of Interest

The authors declare that the research was conducted in the absence of any commercial or financial relationships that could be construed as a potential conflict of interest. The handling editor declared a past co-authorship with the authors.
